# An integrative functional genomics framework for effective identification of novel regulatory variants in genome–phenome studies

**DOI:** 10.1186/s13073-018-0513-x

**Published:** 2018-01-29

**Authors:** Junfei Zhao, Feixiong Cheng, Peilin Jia, Nancy Cox, Joshua C. Denny, Zhongming Zhao

**Affiliations:** 1 Center for Precision Health, School of Biomedical Informatics, The University of Texas Health Science Center at Houston, 7000 Fannin St. Suite 820, Houston, TX 77030 USA; 2 Center for Cancer Systems Biology and Department of Cancer Biology, Dana-Farber Cancer Institute, Harvard Medical School, Boston, MA 02215 USA; 3 Center for Complex Networks Research, Northeastern University, Boston, MA 02215 USA; 4 Vanderbilt Genetics Institute, Vanderbilt University School of Medicine, Nashville, TN 37232 USA; 5 Department of Medicine, Vanderbilt University Medical Center, Nashville, TN 37232 USA; 6 Department of Biomedical Informatics, Vanderbilt University Medical Center, Nashville, TN 37232 USA; 7 Human Genetics Center, School of Public Health, The University of Texas Health Science Center at Houston, Houston, TX 77030 USA

**Keywords:** Phenome-wide association study (PheWAS), Genome-wide association study (GWAS), Regulatory variants, Enhancer, Promoter, Human disease

## Abstract

**Background:**

Genome–phenome studies have identified thousands of variants that are statistically associated with disease or traits; however, their functional roles are largely unclear. A comprehensive investigation of regulatory mechanisms and the gene regulatory networks between phenome-wide association study (PheWAS) and genome-wide association study (GWAS) is needed to identify novel regulatory variants contributing to risk for human diseases.

**Methods:**

In this study, we developed an integrative functional genomics framework that maps 215,107 significant single nucleotide polymorphism (SNP) traits generated from the PheWAS Catalog and 28,870 genome-wide significant SNP traits collected from the GWAS Catalog into a global human genome regulatory map via incorporating various functional annotation data, including transcription factor (TF)-based motifs, promoters, enhancers, and expression quantitative trait loci (eQTLs) generated from four major functional genomics databases: FANTOM5, ENCODE, NIH Roadmap, and Genotype-Tissue Expression (GTEx). In addition, we performed a tissue-specific regulatory circuit analysis through the integration of the identified regulatory variants and tissue-specific gene expression profiles in 7051 samples across 32 tissues from GTEx.

**Results:**

We found that the disease-associated loci in both the PheWAS and GWAS Catalogs were significantly enriched with functional SNPs. The integration of functional annotations significantly improved the power of detecting novel associations in PheWAS, through which we found a number of functional associations with strong regulatory evidence in the PheWAS Catalog. Finally, we constructed tissue-specific regulatory circuits for several complex traits: mental diseases, autoimmune diseases, and cancer, via exploring tissue-specific TF-promoter/enhancer-target gene interaction networks. We uncovered several promising tissue-specific regulatory TFs or genes for Alzheimer’s disease (e.g. *ZIC1* and *STX1B*) and asthma (e.g. *CSF3* and *IL1RL1*).

**Conclusions:**

This study offers powerful tools for exploring the functional consequences of variants generated from genome–phenome association studies in terms of their mechanisms on affecting multiple complex diseases and traits.

**Electronic supplementary material:**

The online version of this article (10.1186/s13073-018-0513-x) contains supplementary material, which is available to authorized users.

## Background

Genome-wide association studies (GWAS) have proven an effective strategy for the detection of variants statistically associated with disease or traits. Since 2005, thousands of single nucleotide polymorphism (SNP)-trait associations have been identified, most of which were deposited in the GWAS Catalog [1]. In recent years, benefiting from the rapid accumulation of detailed phenotypic data from electronic medical records (EMRs), the phenome-wide association study (PheWAS) became feasible as a complementary approach to GWAS to identify genetic susceptibility [2, 3]. Unlike GWAS, in which investigators examine the association of hundreds of thousands to a few million genotypes across the genome with a specific phenotype, PheWAS aims to detect the association of a specific genetic variant with a wide range of physiological and/or clinical outcomes categorized by disease terminologies like the International Classification of Disease (ICD) [4]. One of the advantages of this design is that PheWAS has the ability to identify pleiotropic effects for disease SNPs.

As a proof of concept, the first PheWAS genotyped 6005 European–Americans in Vanderbilt’s biobank using five SNPs that had been previously reported with disease associations in GWAS [4]. After generating case and control populations across all ICD9 code groups for each of these five SNPs, disease-SNP associations were systematically reanalyzed. This study suggested that PheWAS could not only replicate known SNP-disease associations but also identify potentially novel statistical associations. Since this pioneer study, many other groups have applied this strategy to assess previously reported GWAS SNPs and managed to identify new associations and pleiotropic effects [5–7]. In 2013, Denny et al. released the results of the largest PheWAS for that time, namely the PheWAS Catalog, containing 3144 SNPs reported in the GWAS Catalog [8]. However, there are several challenges for PheWAS analysis, such as poor understanding of the functional consequences of variants and potential false positives and false negatives in case assignment. Thus far, appropriate statistical thresholds for defining clinical significance have not yet been reported. Even for the top 202 associations in the PheWAS Catalog, the current estimation of false positive rate for new associations could be as high as 29% [8].

Another challenge is how to improve the interpretation of the associations in the PheWAS Catalog. Previous studies have not systematically examined biological or functional annotations associated with those SNPs. Although one alternative PheWAS approach is to focus on variants with expected function (such as damaging variants with stop-gain and stop-loss) [9], this approach could only be applied to a small proportion of GWAS variants. Furthermore, the majority (~93%) of disease-associated or trait-associated variants discovered in GWAS are located in non-coding sequence [10]. Existing studies have identified a number of such variants involved in transcriptional regulatory mechanisms, including modulation of promoter and enhancer elements and enrichment within expression quantitative trait loci (eQTLs) [11–14]. Previous studies have suggested that there was significant enrichment in functional SNPs among the currently identified association results in the GWAS Catalog [10, 15, 16]. Thanks to the recent advances of functional genomics studies, several national and international projects, such as FANTOM5 [17], ENCODE [18], NIH Roadmap [19], and GTEx [20], have generated massive amounts of functional data in various human cell lines or tissues. Comprehensive investigation of the functional or regulatory roles of the variants reported by PheWAS and further investigation of their tissue-specific regulatory networks will be important for our deeper understanding of the biological consequences of the significant SNPs involved in various complex diseases or traits.

**Fig. 1:**
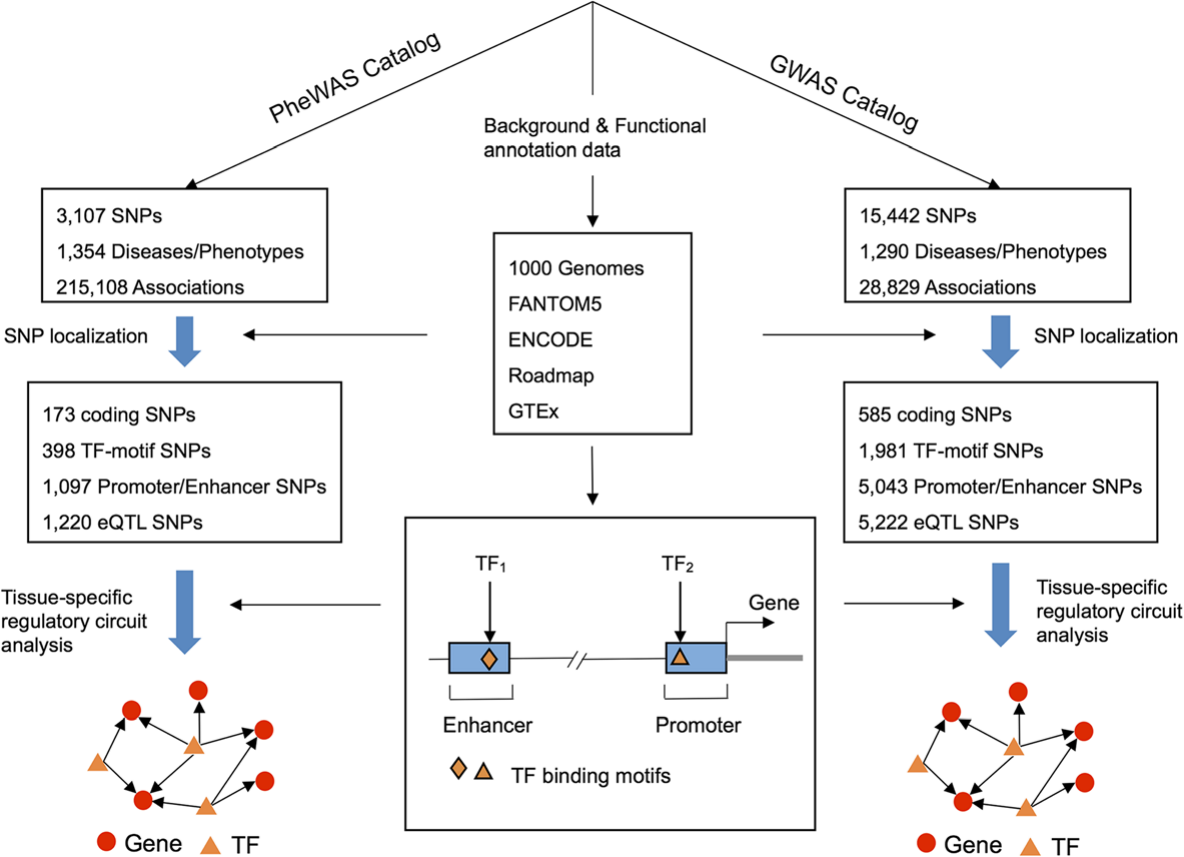
*Diagram* of an integrative functional genomics workflow. SNPs from the PheWAS Catalog and GWAS Catalog were mapped to the whole human genome and non-coding SNPs were re-annotated with regulatory information. Protein-coding SNPs were re-annotated with protein functional information, including protein–ligand binding sites and phosphorylation sites. Based on gene regulatory annotations, we also performed a tissue-specific regulatory circuit analysis. All detailed data are provided in Additional files 1–5: Tables S1–S5

In this study, we performed a comprehensive investigation of the functional regulation of variants derived from the PheWAS Catalog through an integrative functional genomics framework (Fig. 1). Specifically, we incorporated functional annotation data, including transcription factor (TF)-motif, promoter, enhancer, and eQTL information from FANTOM5, ENCODE, NIH Roadmap, and GTEx, into 215,108 significant SNP-trait associations connecting 3107 SNPs and > 1000 complex diseases or traits collected in the PheWAS Catalog. We found a significant enrichment of functional SNPs in these disease-associated loci in PheWAS compared to the polymorphisms generated from the 1000 Genomes (1KG) project, which is comparable to disease associated loci in the GWAS Catalog. We further constructed tissue-specific gene regulatory networks, namely TF-promoter/enhancer-target gene networks, to examine the tissue-specific regulatory circuits for the significant SNP-trait association results in the PheWAS Catalog. We found that functional annotations significantly improved the power of detecting novel associations in the PheWAS Catalog. Furthermore, we found that dozens of novel associations in the PheWAS Catalog had strong functional evidence even though they only exhibited moderate significance, often likely due to inadequate sample size in the original study. Finally, we constructed the tissue-specific regulatory circuits for several complex traits, such as mental diseases and autoimmune diseases in case studies. In summary, this study sheds light on the functional consequences of disease-associated loci and it offers a powerful approach to identify novel SNP-trait associations in PheWAS.

## Methods

### SNP annotations

We downloaded all the SNP-phenotype association results from the GWAS Catalog [1] (September/2015) and the PheWAS Catalog [8] (October/2015). We first annotated each SNP with transcription information from RefSeqGene using ANNOVAR [21]. We further mapped the protein-coding SNPs onto protein structures and identified those SNPs affecting protein functional sites: protein–ligand binding sites and phosphorylation sites. Then, we annotated the remaining non-coding SNPs with three types of genomic functional information: motif; promoter/enhancer; and eQTL, respectively. Single nucleotide variants (SNVs) from the 1000 Genomes project were also annotated in the same way. We then performed Fisher’s exact test on a 2 × 2 table to calculate a *P* value for the difference in the frequency of functionally annotated SNPs between all the reported SNPs and the SNVs from the 1000 Genomes project.

### Proteins’ structural genomics data

We collected two types of proteins’ functional site information: ligand-binding sites and phosphorylation sites. We extracted protein–ligand binding site data from BioLiP, which is a semi-manually curated database for high-quality, biologically relevant protein–ligand binding interactions [22]. For each UniProt protein, we combined the protein–ligand binding site residues of all the corresponding PDB structures. In total, there were 17,595 UniProt proteins with protein–ligand binding site information. We mapped all protein-coding SNPs generated from PheWAS and GWAS as described in our previous study [23–25]. We also collected human phosphorylation site information from the PhosphoSitePlus [26] and dbPTM3 databases [27]. The detailed data preparation for phosphorylation sites was described in our previous studies [28, 29]. In total, we obtained 173,460 non-redundant phosphorylation sites on 18,610 proteins.

### Genome-wide functional annotation data

We collected three types of functional annotation information: motif, promoter/enhancer, and eQTL. Motif data were extracted from the ENCODE-motif that was available from the MIT Computational Biology Group (http://compbio.mit.edu/encode-motifs/). In total, we collected the position information of 1772 motifs for 662 TFs. Promoter/enhancer information was obtained from FANTOM5 (http://fantom.gsc.riken.jp/data/), Roadmap (http://egg2.wustl.edu/roadmap/web_portal/), and ENCODE (through UCSC Genome Browser [30]). We downloaded eQTL analysis results of 44 tissues from the GTEx V6 release (http://www.gtexportal.org/). In the GTEx analysis, cis-eQTLs were calculated for all the SNPs within ± 1 Mb of the transcriptional start site (TSS) of each gene. Each eQTL is defined as a SNP being significantly *cis-*associated with the expression difference of at least one gene by false discovery rate (FDR) ≤ 0.05. SNVs from the final phase of the 1000 Genomes project were retrieved from the EBI FTP Site in VCF format (http://ftp.1000genomes.ebi.ac.uk/vol1/ftp/release/20130502/).

### Linkage disequilibrium

We used the online SNP Annotation and Proxy (SNAP) tool (https://www.broadinstitute.org/mpg/snap/) to search for the proxy SNPs of each reported SNP in the PheWAS Catalog and GWAS Catalog based on linkage disequilibrium (LD), confined to the CEU population of HapMap 3 (release 2). For each of the reported SNPs, we obtained those SNPs that were in its strong LD (*r*^2^ ≥ 0.8) within the 500-kb flanking region of each side (upstream and downstream) of the SNP.

### Tissue-specific regulatory circuit analysis

We downloaded 394 cell type-specific and tissue-specific regulatory networks from http://regulatorycircuits.org/. For our analysis, if the SNPs were in the promoter sequences or promoter’s 400-bp upstream to 50-bp downstream sequences, they would be considered possibly affecting promoter function. We considered SNPs that possibly affected enhancers if they were located in the enhancer sequences. The detailed description is provided in a recent study [31].

### Tissue specificity of gene expression

We downloaded the gene expression data of 32 tissues from GTEx V6 release (http://www.gtexportal.org/). For each tissue, we regarded those genes with RPKM ≥ 1 in > 80% samples as tissue-expressed genes and the remaining genes as tissue-unexpressed. To quantify the expression significance of tissue-expressed gene *i* in tissue *t*, we calculated the average expression 〈*E*(*i*)〉 and the standard deviation *δ*_*E*_(*i*) of a gene’s expression across all considered tissues [32]. The significance of gene *i* in tissue *t* is defined as

1 $$ {z}_E\left(i,t\right)=\left(E\left(i,t\right)-\left\langle E(i)\right\rangle \right)/{\delta}_E(i) $$

### Collection of disease-associated genes

Disease-associated genes were collected from DisGeNET v4.0 [33]. We used all the 429,036 gene-disease associations that covered 17,381 genes and 15,093 diseases, disorders and clinical or abnormal human phenotypes. Fisher’s exact test was used to calculate *P* values for the enrichment of disease genes among the perturbed modules obtained from functional annotation and the raw PheWAS data.

### Statistical analysis and network visualization

All the statistical analyses were performed using R v3.2.3 (http://www.r-project.org/). We illustrated the network graphs using Cytoscape (v2.8.1) [34].

## Results

### An integrative functional genomics framework

We developed an integrative functional genomics framework to examine the functional regulation and tissue-specific regulatory circuits for large-scale disease-associated SNPs reported in the GWAS Catalog and PheWAS Catalog (Fig. 1). To examine the regulatory roles of variants in the PheWAS Catalog, we downloaded data from http://phewascatalog.org, which included 215,108 significant disease-SNP associations (*P* < 0.05) connecting 1354 disease terms and 3107 SNPs. As a comparison, we downloaded data from the GWAS Catalog (data downloaded on 27 April 2015), including 28,829 significant disease-SNP associations (*P* < 1.0 × 10^–5^) connecting 1290 disease terms or traits and 15,442 SNPs from 2153 published papers. Then, we performed systematic localization for the disease SNPs in various functional regions including TF-motifs, promoters, enhancers, and eQTLs based on the data from FANTOM5 [17], ENCODE [18], NIH Roadmap [19], and GTEx [20]. We used SNP data from the 1000 Genomes project as background in our enrichment analysis of the disease SNPs in various functional regions [35]. After functional annotation, we constructed tissue-specific gene regulatory networks (TF–Promoter/Enhancer–Target gene) and investigated the associations in the PheWAS Catalog at the tissue-specific regulatory circuit level (Fig. 1).

### Functional atlas of variants generated from PheWAS

First, we annotated each SNP with RefSeq gene information using ANNOVAR [21]. From the PheWAS Catalog, we found 173 SNPs (5.5%) in exon regions. This result is consistent with a previous report that most (~93%) disease SNPs in the GWAS Catalog are in non-coding regions [10]. Further analysis showed two SNPs located at ligand-binding sites or within their two-residue flanking regions (rs1800961, *HNF4A*: p.T139I; and rs1057910, *CYP2C9*: p.I359L, Additional file 1: Table S1) and 15 SNPs at phosphorylation sites or within their seven-residue flanking regions (e.g. rs1801275, *IL4R*: p.Q576R; and rs11906160, *MYH7B*: p.A25T, Additional file 2: Table S2). *CYP2C9*: p.I359L is reported to be related with deep vein thrombosis by PheWAS and with warfarin maintenance dose by GWAS [36]. However, individuals who carry CYP2C9: p.I359L are poor metabolizers and require lower doses of warfarin to achieve similar anticoagulation. Note that other variants in CYP2C9 are candidate factors in different warfarin dosing. While the finding here unveiled the possible underlying functional role of this SNP, much more functional and pharmacological work will be needed for more evidence for this SNP in warfarin dosing.

Among the 585 exonic SNPs from the GWAS Catalog, there were eight SNPs located at ligand-binding sites or within their two-residue flanking regions (Additional file 3: Table S3) and 45 SNPs at phosphorylation sites or within their seven-residue flanking regions (e.g. rs7412, APOE: p.R176C, Additional file 4: Table S4). These ligand-biding site SNPs are rs1057910, CYP2C9: p.I359L; rs16844401, HGFAC: p.R516H; rs9381199, UBR2: p.T154I; rs1229984, ADH1B: p.H48R; rs1303, SERPINA1: p.E400D; rs5880, CETP: p.A390P; rs1800961, HNF4A: p.T139I; and rs1058172, CYP2D6: p.R365H. Apolipoprotein-E (apoE) is important in neuronal lipid transport and is thought to stabilize microtubules by preventing tau hyperphosphorylation [37]. The GWAS Catalog also reported the association between SNP rs7412 and lipid metabolism phenotypes [38]. These analyses revealed that protein-coding SNPs with putative functions (e.g. altering ligand–protein binding sites and phosphorylation sites) only accounted for approximately 3% of the total SNPs in the GWAS Catalog. We next systematically investigated the regulatory information for the remaining 97% non-coding variants using an integrative functional genomics framework as illustrated in Fig. 1.

**Fig. 2:**
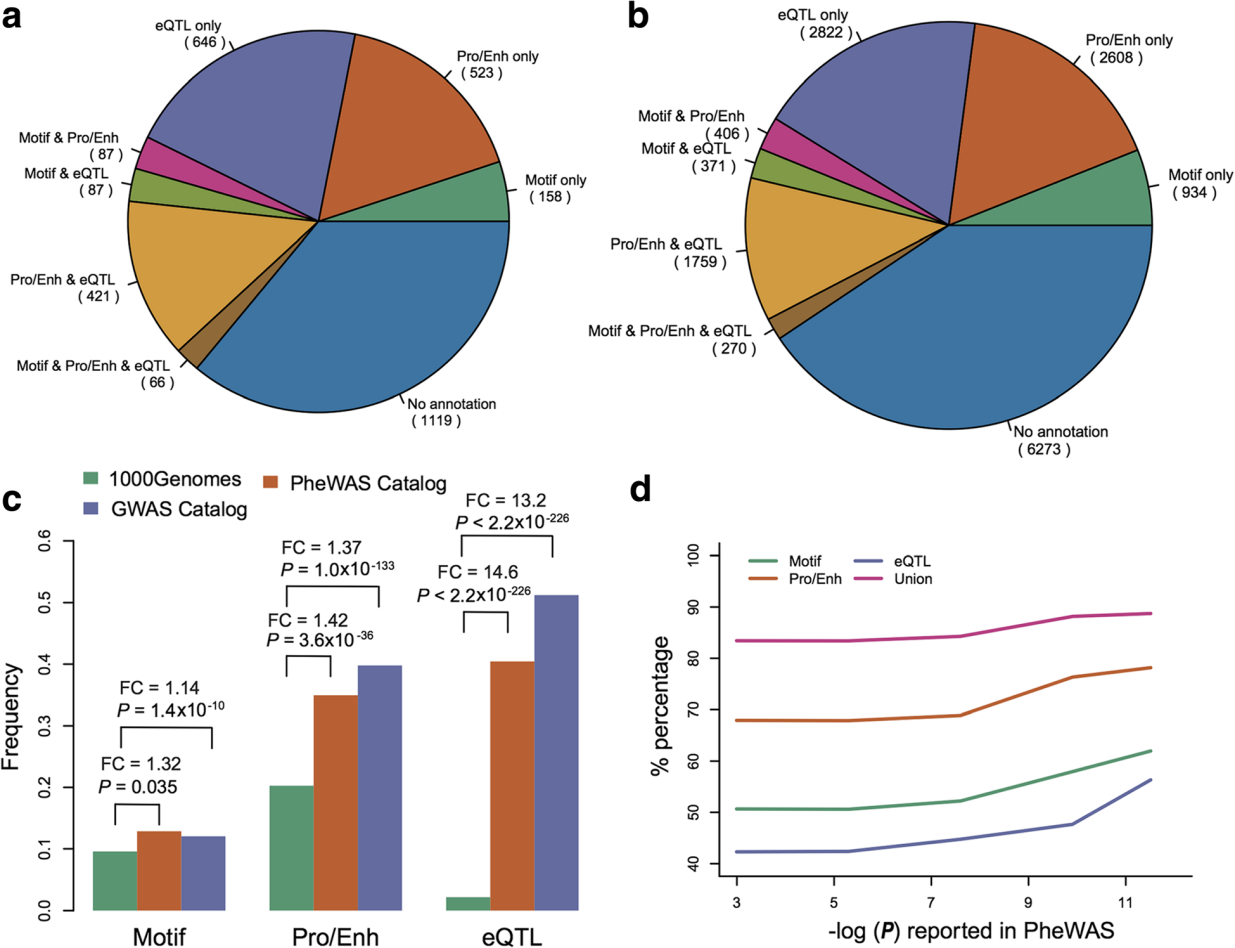
SNP annotation and enrichment analysis in different types of functional data. **a** Proportions of SNPs from the PheWAS Catalog in different types of functional data. **b** Proportions of SNPs from the GWAS Catalog in different types of functional data. **c** Enrichment analysis for different types of functional data with the variants from the 1000 Genomes project as the background. FC fold-change. **d** Proportions of SNPs in different types of functional data after linkage disequilibrium (LD) extension by the *P* value reported in the PheWAS Catalog

Overall, > 60% of the non-coding SNPs in the PheWAS Catalog could be annotated with one of the functional categories: TF-motif, promoter/enhancer, or eQTL (Fig. 2a and Additional file 5: Table S5). Specifically, 398 SNPs (12.6%) were found to be located in the motif regions of at least one TF, 1097 SNPs (34.8%) overlapped with a promoter/enhancer detected in at least one cell line, and 1220 SNPs (38.8%) were eQTLs in at least one tissue type in GTEx. There were 859 SNPs (27.3%) that could be annotated with more than one type of functional category and 66 SNPs (2.1%) that had functional support from all three types of information. The enrichment of functional annotations for these SNPs suggested that the PheWAS SNPs might play important roles in disease or traits through functional regulation. The detailed annotated data for regulatory variants in the PheWAS Catalog is provided in Additional file 5: Table S5. A similar distribution was observed for the GWAS Catalog (Fig. 2b). Specifically, there were 1981 SNPs (12.8%) located in the motif regions of at least one TF, 5043 SNPs (32.6%) overlapped with a promoter/enhancer of at least one cell line, 5222 SNPs (33.8%) with an eQTL in at least one tissue type in GTEx, 2806 SNPs (18.1%) that could be annotated with more than one type of functional information, and 270 SNPs (1.7%) that had functional support from all three types of information.

Interestingly, a previous study has suggested that a functional SNP with the strongest experimental evidence is often not the reported SNP itself in GWAS; rather, it is a SNP within LD of the reported SNP [15]. Inspired by this finding, we next examined the proxy SNPs that were in strong LD (*r*^2^ ≥ 0.8) with the reported SNP in the CEU population (Utah residents with Northern and Western European ancestry from the CEPH collection) of HapMap 3 (release 2) [39]. We performed LD extension analysis: (1) for each reported SNP, we located and annotated its proxy SNPs with the same regulatory information; and (2) a reported SNP was also considered as annotated if one of its proxy SNPs could be annotated with functional information even if the reported SNP itself was not in the functional region. After LD extension, the frequency of annotated SNPs reached up to 80%. When considering the annotated SNPs supported by more than one type of functional information, the frequency increased to 55.6% from 27.3%. For example, 686 reported SNPs (21.8%) or their proxy SNPs had functional evidence from all three types of information. Next, we performed the same analysis on the full SNP set from the GWAS Catalog and found that the SNPs in the GWAS Catalog displayed a similar trend (Additional file 6: figure S1).

### PheWAS variants are enriched in functional regions

We used the SNVs from the 1000 Genomes project as the background to assess the significance of the enrichment of functional elements among the disease-associated SNPs in the PheWAS Catalog and GWAS Catalog, respectively. We found a significant overall enrichment for regulatory functions in PheWAS disease-associated SNPs. Similar trends in the GWAS Catalog were observed. Figure 2c shows the enrichment analysis for different types of functional data. In comparison with the 1000 Genomes SNPs, we observed weak enrichment for TF-motif (1.32-fold, *P* = 0.035, Fisher’s exact test), moderate enrichment for promoter/enhancer (1.42-fold, *P* = 3.6 × 10^–36^), and strong enrichment for eQTLs (14.6-fold, *P* < 2.2 × 10^–226^), respectively, in the PheWAS Catalog. Similarly, comparison of the GWAS Catalog with the 1000 Genomes data revealed 1.14-fold enrichment of TF-motif (*P* = 1.4 × 10^–10^), 1.37-fold enrichment for promoter/enhancer (*P* = 1.0 × 10^–133^), and 13.2-fold enrichment for eQTLs (*P* < 2.2 × 10^–226^), respectively (Fig. 2c).

We also observed an interesting phenomenon that the enrichment of functional elements was positively correlated with the statistical power of the initial SNP-phenotype association in the PheWAS Catalog. This implies that those SNPs involved in associations with stronger statistical power may be more likely to be functional (*P* = 0.015, Pearson’s correlation, Fig. 2d). This is consistent with the previous observation that the likelihood of PheWAS replicating a GWAS Catalog association is directly related to the statistical power of the initial SNP-phenotype association [8].

### Re-identifying novel associations in PheWAS

**Fig. 3:**
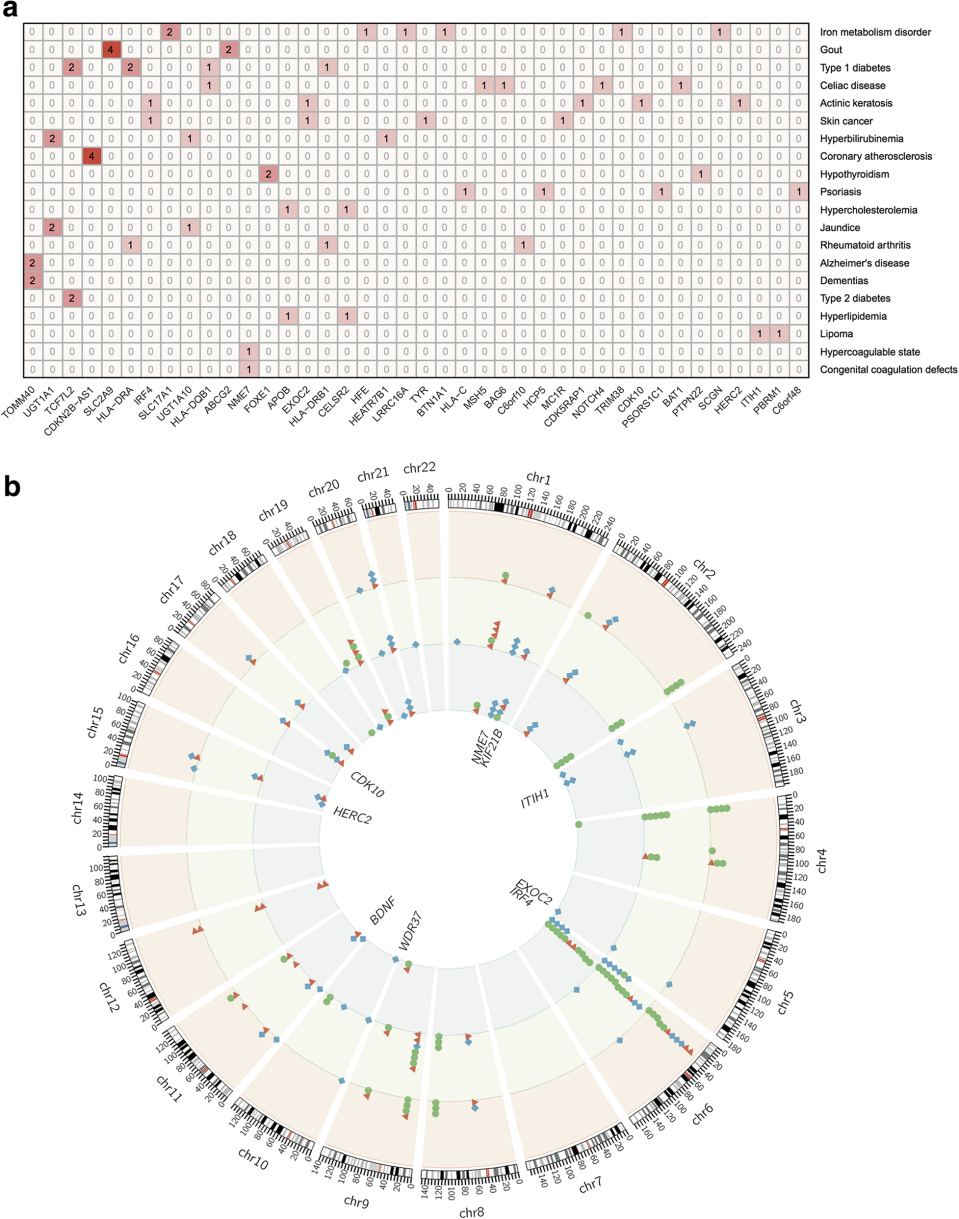
Overview of PheWAS associations in the genome after functional annotation. **a** This matrix shows the number of functional SNPs for their respective phenotype. **b** The *Circos plot* showing the PheWAS associations in different types of functional data. *Red triangles* represent the associations in the GWAS Catalog only, *green circles* represent GWAS Catalog associations replicated by PheWAS (*P* < 0.05), and *blue diamonds* represent new phenotype associations identified by PheWAS (*P* < 4.6 × 10^−6^ or FDR < 0.1)

In order to search for novel associations, Denny et al. used a FDR < 0.1 (*P* < 4.6 × 10^–6^) and detected 202 associations for 102 SNPs and 87 phenotypes [8]. Focusing on these 102 SNPs, we found that 61.8% of them (63/102) were eQTLs in at least one tissue type in GTEx and 89.2% of them (91/102) could be annotated with at least one type of functional information after LD extension. Among these 87 phenotypes, 63 (31%) were categorized to be potentially novel associations by FDR < 0.1. For example, for two mental disorder-related functional SNPs near genes *PBRM1* and *ITIH1* in the GWAS Catalog, PheWAS suggested they might be associated with lipoma. In addition, 109 (54%) were either replications or associations with phenotypes related to associations in the GWAS Catalog. For example, PheWAS replicated the associations for four functional SNPs in the gene *CDKN2B − AS1* to coronary atherosclerosis. Figure 3a shows these associations on the background of functional annotation for 20 selected diseases or traits.

### Discovery of tissue-specific regulatory circuits altered by PheWAS variants

Although integration of functional annotation data is a promising strategy in prioritizing and fine-mapping disease variants in the PheWAS Catalog, it overlooks the interplay between variants at the cellular level. This problem can be partially addressed when we examine them at the biological pathway and regulatory network levels. Multiple previous studies have applied pathway-based and network-based approaches to identify pathways or network modules based on the connectivity between disease-related genes, but the networks they relied on were typically protein–protein interaction, [40–42], gene co-expression [43–45], or functional association networks [46], which lack detailed regulatory information and tissue-specific information.

**Fig. 4:**
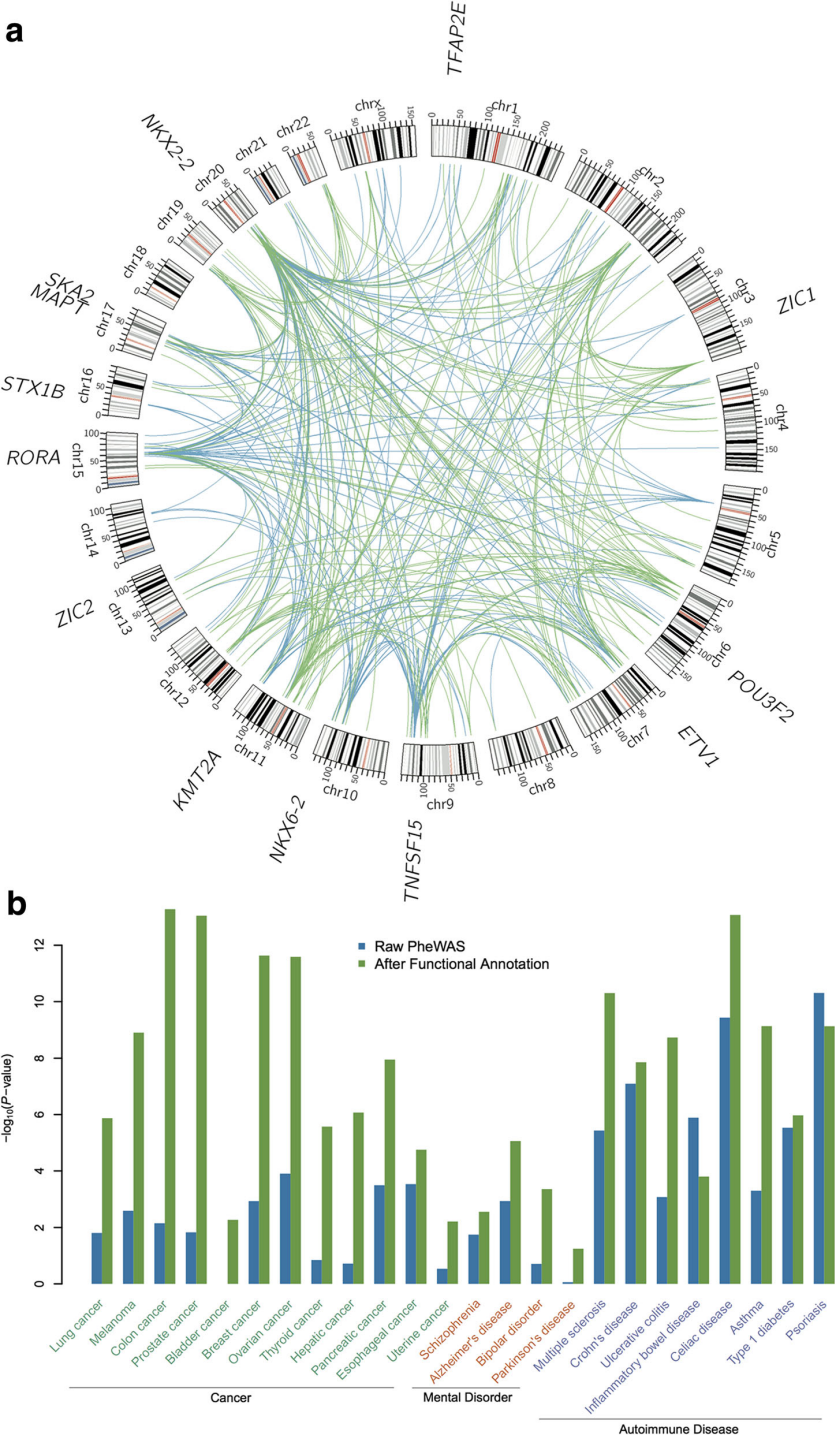
*Illustration* of tissue-specific TF-promoter/enhancer-target gene interaction network analysis. **a** Overview of the perturbed tissue-specific TF-target gene network in mental disorders. *Blue links* represent enhancer-perturbed TF-target gene interactions. *Green links* represent promoter-perturbed TF-target gene interactions. Several disease-associated genes are highlighted in the outside of the *circle*. **b** Enrichment analysis of the disease genes in the perturbed tissue-specific TF-target gene network

Here, we mapped the genotype–phenotype association results in PheWAS onto their respective tissue-specific regulatory circuits. The tissue-specific regulatory circuits were built based on three components: (1) genome-wide mapping of promoters and enhancers; (2) linking TFs to promoters and enhancers; and (3) linking enhancers and promoters to target genes based on data from the FANTOM5 consortium, as described in a previous study [31]. For each disease, we reconstructed the perturbed disease-relevant subnetwork with two types of edges: (1) enhancer-perturbed TF-target interaction if one disease-associated SNP affects TF-enhancer binding; and (2) promoter-perturbed TF-target interaction if one SNP affects TF-promoter binding. We illustrated this using mental disorders (Fig. 4a). To validate the relationship of these perturbed modules with disease, we performed disease-associated gene enrichment analysis using the data from DisGeNET v4.0, which included 429,036 gene-disease associations comprising 17,381 genes and > 15,000 diseases and phenotypes [47]. As a comparison, we extracted the nearest genes of the significant SNPs in the original PheWAS Catalog to perform the same enrichment analysis. The results showed that most of these perturbed modules were more significantly enriched with disease-associated genes when compared with the results by using the nearest gene of each SNP (Fig. 4b). In the following section, we describe the novel tissue-specific regulatory circuits identified for three types of complex diseases: cancer; brain-related diseases; and autoimmune diseases, as case studies.

### Identifying new tissue-specific regulatory circuits for breast cancer

For breast cancer, we identified two significant SNPs involved in TF targeting promoter/enhancer with strong confidence by altering gene expression in disease-associated genes. One interesting discovery is SNP rs242557, which was found to be associated with progressive supranuclear palsy from the GWAS Catalog (*P* = 9 × 10^–18^) [48]. While in the PheWAS Catalog, this SNP is reported to be associated with breast cancer and schizophrenia. Based on functional annotation, we found that SNP rs242557 was located in a motif-enriched enhancer region and it regulates the expression of genes *MAPT* and *CRHR1. MAPT*, encoding the microtubule-associated protein tau that binds and stabilizes microtubules, plays an important role in neuronal polarity and signal transduction. Mutations on *MAPT* have been associated with several neurodegenerative disorders such as Alzheimer’s disease (AD), Pick’s disease, frontotemporal dementia, cortico-basal degeneration, and progressive supranuclear palsy [49]. In cancer-related studies, it has been shown that low tau expression renders microtubules more vulnerable to paclitaxel and makes breast cancer cells hypersensitive to paclitaxel [50].

Another example is SNP rs6478109 located in the promoter region of *TNFSF15*. This SNP may affect the binding motif of multiple transcriptional factors and lead to the dysfunction of *TNFSF15*. A clinical investigation has indicated that high levels of *TNFSF15* were associated with increased survival rates of breast cancer patients [51, 52]. We also identified several SNPs where their LD regions had more evidence supporting a regulatory role than the SNPs themselves. For example, SNP rs2885805 is reported to be associated with cytomegalovirus antibody response in the GWAS Catalog [53]. There has been no reported evidence that supports the functional role of SNP rs2885805 itself. Here, we found that it was in strong LD (r^2^ = 0.857) with SNP rs2885805, a functional SNP located in the enhancer region of *CD53*, which is a prognostic gene signature in breast cancer [54, 55]. A recent study revealed that elevation in serum cytomegalovirus immunoglobulin antibody levels preceded the development of breast cancer in some women [56], suggesting the potential biological implication of this observation and the potential link between cytomegalovirus antibody response and breast cancer. Further functional validation is needed to conform this association.

### Identifying tissue-specific regulatory circuits for brain-related diseases

We next examined the tissue-specific regulatory circuits (TF-target gene regulatory network) for four brain-regulated diseases: schizophrenia; AD; bipolar disorder; and Parkinson’s disease. Figure 4b shows that SNPs with functional annotation had lower *P* values in PheWAS for all four brain-related diseases. For schizophrenia, one novel associated SNP identified by PheWAS is SNP rs339969 with *P* = 0.046 based on 60 cases. In the GWAS Catalog, it is reported that this is associated with Gamma-glutamyl transferase from a previous study [57]. Functional annotation indicates that SNP rs339969 is located in a motif-enriched enhancer whose target genes include *RORA*. The retinoic acid-related orphan receptor alpha (RORA), which is a ligand-dependent orphan nuclear receptor, acts as a transcriptional regulator and has been previously identified as a novel candidate gene for autism spectrum disorders [58].

**Fig. 5:**
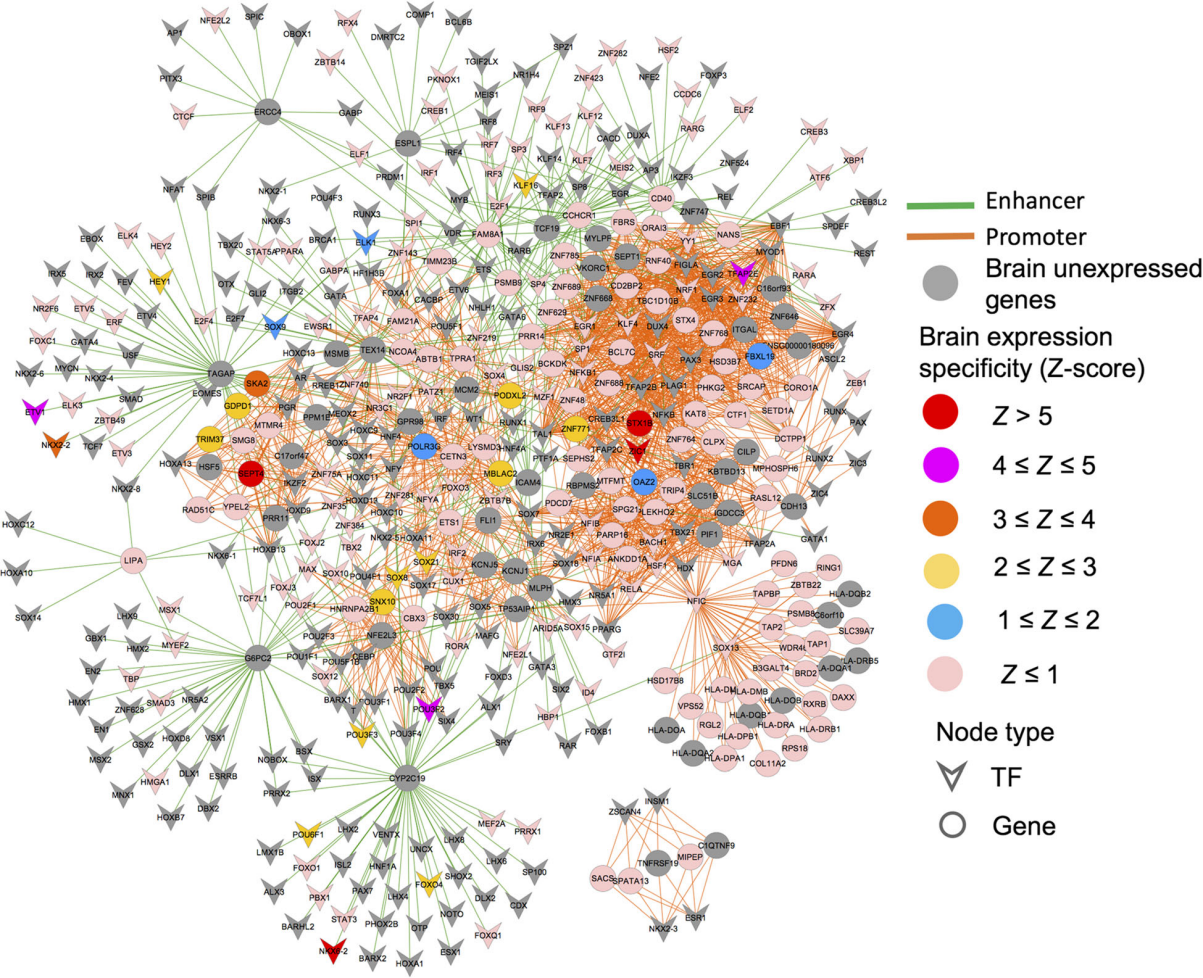
A brain-specific TF-target gene regulatory network for AD. The TF-target gene network was generated by mapping the significant SNPs with AD from the PheWAS Catalog into the enhancer or promoter regions via three components: (1) genome-wide mapping of promoters and enhancers; (2) linking TFs to promoters and enhancers; and (3) linking enhancers and promoters to target genes as described in “Methods.” TFs are denoted by Vee and target genes with significant SNPs are denoted by *circles*. The node color is coded based on the brain-specific gene expression quantified by z-scores using the RNA-sequencing (RNA-seq) data from GTEx (see “Methods”). A larger z-score indicates a higher expression level in brain compared to other tissues. *Green lines* represent the enhancer-gene regulations and *orange lines* represent the promoter-gene regulations. Several TFs and targeted genes (e.g. *ZIC1*, *STX1B*, *CDH13*, and *SKA2*) described in the main text are *highlighted*. Both Figs. 5 and 6 were prepared using Cytoscape (v2.8.1)

We next built a brain-specific TF-target gene regulatory network for AD as shown in Fig. 5. The brain expression specificity for each gene was calculated by Z-score using 1632 brain samples compared to 10,346 samples across other 31 tissues from GTEx (see “Methods”). A lower Z-score means higher brain-specific expression. SNP rs7197475 is associated with systemic lupus erythematosus in the GWAS Catalog [59]. However, PheWAS suggests it is associated with AD based on 737 cases (*P* = 0.015). Although there is no functional evidence for this association from the annotation of this SNP itself, we identified another SNP rs7194347, in perfect LD (r^2^ = 1) with SNP rs7197475, which strongly supports this discovery. SNP rs7194347 overlaps with an enhancer region and may perturb the expression of *STX1B* whose methylation and expression changes are associated with Parkinson’s disease [60]. *STX1B* showed highly brain-specific expression with its Z-score = 5 when compared to the other 31 tissues, suggesting a potential functional gene for AD (Fig. 5). Another similar example is SNP rs2302189 whose association with dental caries is reported in the GWAS Catalog. We identified a SNP rs9898916 in strong LD (r^2^ = 0.87) with this SNP. SNP rs9898916 is involved in the regulation of *SKA2* whose methylation is associated with decreased prefrontal cortical thickness and greater post-traumatic stress disorder (PTSD) severity among trauma-exposed veterans [61]. In addition, epigenetic variation at *SKA2* mediates vulnerability to suicidal behaviors and PTSD through dysregulation of the hypothalamic pituitary adrenal axis in response to stress [62], suggesting potential biological implication of *SKA2* in PTSD. Two previous studies have reported that the *ZIC1* gene encodes a TF that binds and trans-activates the apolipoprotein E gene and further plays an important role in neuronal maintenance and repair [63, 64]. GTEx data show that *ZIC1* is highly expressed in brain (Fig. 5). In our analysis, we identified one proxy SNP (dbSNP ID: rs4783244) that is considered as being AD-related by altering the binding motif of *ZIC1* and perturbing the expression of the target gene *CDH13. CDH13* encodes T-cadherin, a GPI-anchored protein with cell adhesion properties that is highly expressed in the brain and cardiovascular system. A previous study suggested that *CDH13* might be a promising candidate gene for attention deficit hyperactivity disorder (ADHD) [65].

### Identifying tissue-specific regulatory circuits for autoimmune diseases

**Fig. 6:**
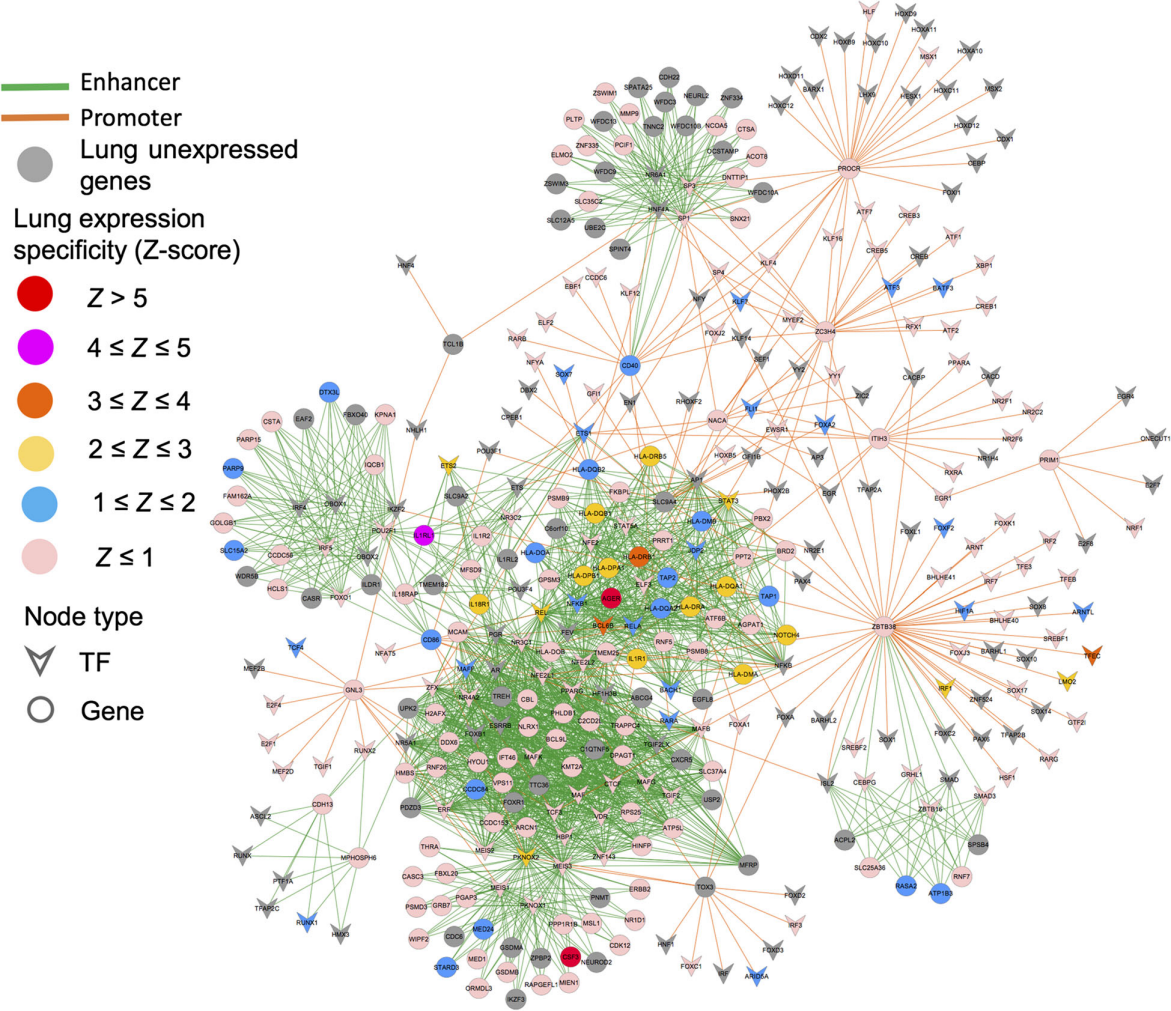
A lung-specific TF-target gene regulatory network for asthma. The TF-target gene network was generated by mapping the significant SNPs with asthma from the PheWAS Catalog into the enhancer or promoter regions via three components: (1) genome-wide mapping of promoters and enhancers; (2) linking TFs to promoters and enhancers; and (3) linking enhancers and promoters to target genes as described in “Methods.” TFs are denoted by Vee and target genes with the significant SNPs are denoted by *circles*. The node color is coded based on the lung-specific gene expression quantified by z-scores using the RNA-seq data from GTEx. A larger z-score indicates a higher expression level in lung compared to other tissues. *Green lines* represent the enhancer-gene regulations and *orange lines* represent the promoter-gene regulations. Several TFs and targeted genes (*CSF3*, *ZBTB38*, *NFKB1*, *HLA-DRB1*, *HLA-DPB1*, and *HLAnDRB5*) described in main text were highlighted

We further built a lung-specific TF-target gene regulatory network for asthma in Fig. 6. The lung expression specificity for each gene was calculated by Z-score using 497 lung samples compared to 11,973 samples across the other 31 tissues from GTEx (see “Methods”). A lower Z-score means higher lung-specific expression. In an autoimmune disease analysis (Fig. 4b), PheWAS reported an association between enhancer SNP rs6763931 (located in an intron of *ZBTB38*) and asthma. Later this was confirmed by one GWA study [66]. In total, there were four genes (*RASA2*, *ZBTB38*, *RNF7*, and *SLC25A36*) in a 1-Mb region centered by SNP rs6763931. Functional evaluation showed that *RASA2* (103 Kb away from SNP rs6763931) was highly differentially expressed between children with asthma and healthy individuals while the host gene (*ZBTB38*) showed no evidence of differential expression [66]. SNP rs1420101 (located in *IL1RL1*) is a variant affecting the quantity of eosinophil in pleiotropic multifunctional leukocytes, which is involved in inflammatory and immune responses observed in asthma, eczema, rhinitis, and other inflammatory diseases [67]. PheWAS confirmed the association of SNP rs1420101 with asthma based on 1390 cases (*P* = 0.0015).

The major histocompatibility complex (MHC) is one of the most variable and gene-dense regions in the human genome with potential effects on innate and specific immunity. In the MHC region, PheWAS reported an association between asthma and SNP rs660895. This SNP may alter the binding enhancer of *NFKB* whose target genes include *HLA-DRB1*, *HLA-DRB5*, and *HLA-DPB1* (Fig. 6). The association between asthma and the *HLA-DRB1* locus has been identified in a family-based population sample [68]. In addition, our gene regulatory network analysis is consistent with a recent PheWAS with HLA variants [69].

While PheWAS replicated the association between SNP rs2305480 and asthma in the GWAS Catalog, our functional annotation suggested another SNP rs9909593 that is in perfect LD with SNP rs2305480. SNP rs9909593 might be involved with the TF PKNOX1 that regulates *CSF3* (encoding Colony Stimulating Factor 3). *CSF3* is a protein-coding gene that is important for the survival and proliferation for neutrophils and macrophages. GTEx data showed that *CSF3* is a lung-specifically expressed gene with Z-score = 5 (Fig. 6). This indicates its important regulatory role in lung function. A previous study reported that genetic variation on *CSF3* was associated with cross-sectionally measured lung function in smokers [70].

## Discussion

Understanding the genetic architecture of disease can help elucidate relevant biochemical pathways for drug targets and enable personalized medicine. Toward this direction, both GWAS and PheWAS have been successful in identifying thousands of disease-variant associations for further studies. Most of these disease-associated variants are located in non-coding regions and exert regulatory roles in modulating the expression of downstream target genes. In this study, we performed functional annotations of the regulatory variants in both the PheWAS Catalog and GWAS Catalog. Our functional annotation analysis demonstrated that both the PheWAS and GWAS significant variants are enriched within regulatory regions in the human genome, from which putative functional mechanisms for these associations can be further explored and validated. While no large GWAS-PheWAS datasets are currently available for a systematic validation of our findings, here we showcased functional validation for the identified associations in inflammatory bowel disease (IBD) on the colon-specific TF-target gene regulatory network. We found that one of the new network-predicted IBD genes in our reconstructed colon-specific TF-target gene regulatory network (Additional file 6: figure S2), *MAFB*, was validated by very recently published functional data in macrophages [71]. However, much more functional work is needed to validate the identified associations via in vitro or in vivo assays in order to fully unveil the underlying regulatory mechanisms.

Together, this systematic investigation revealed that gene regulation plays important roles for significant trait-SNP associations derived from the PheWAS Catalog, which is comparable with the GWAS Catalog. In addition, our results demonstrated similar distributions of SNP functionality in the PheWAS catalog and the current GWAS Catalog. This is not surprising in consideration that the PheWAS Catalog set we chose was originally derived from the GWAS Catalog as of 2012, but exploring functional roles of the SNPs in multiple phenotypes currently remains an important task.

In PheWAS, large-scale multiple testing is needed to control the FDR. However, standard FDR control procedures, such as the Benjamini–Hochberg procedure [72], are typically built on the assumption of independence and would fail to provide optimal power when the individual tests are strongly correlated and differ in statistical properties such as sample size, true effect size, signal-to-noise ratio, or prior probability of being false as in the PheWAS setting [73, 74]. Recently, there have been several studies focused on how to use a data-driven hypothesis-weighting strategy to improve the detection power of large-scale multiple testing [73, 74]. The results in our analysis suggests that functional annotation may be a good choice in weighting associations in the PheWAS Catalog. In addition, our analysis demonstrated that integrating regulatory information for variants in PheWAS dramatically improved the power to identify previously published disease-associated genes derived from DisGeNET v4.0 [33] (Fig. 4b), providing complementary evidence that will not only strengthen previously identified associations but also enhance the discovery of new sets of causal genes for complex diseases. However, potential literature bias and data incompleteness of disease-associated genes in DisGeneNET may influence the current enrichment analysis. In our study, we used *P* < 1.0 × 10^–5^ to include more potential SNP-trait association pairs while reasonably controlling false positive rate. If we used *P* < 5.0 × 10^–8^ as the cutoff, there would be not enough SNPs for follow-up analyses, though the conclusion could remain the same. Our rationale is that moderate association signals can be useful in integrative bioinformatics analyses in order to identify more functional candidates (e.g. network) for follow-up validation. This strategy has been demonstrated as being effective in previous studies [75, 76]. Taken together, regulatory analysis could prove an important addition to many upcoming PheWAS and GWAS, especially for the studies without large population sizes.

Reported results in multiple GWASs have highlighted a number of pleiotropic effects. Compared to GWAS, one promising advantage of PheWAS is to examine pleiotropy by measuring genetic associations of one variant with thousands of diseases or phenotypes simultaneously. Variants demonstrating pleiotropy may confer tissue-specific effects on multiple genes [77], some of which could occur on different chromosomes (trans-effects [78]). Examination of expression data in a relevant tissue type could help identify the tissue-specific regulatory changes caused by each variant [79, 80], as demonstrated in the GTEx project [20]. In our analysis, we also observed the tissue-specific expression profile of the same target gene for one disease-associated SNP with pleiotropic effects revealed by PheWAS. This may indicate a promising role of a tissue-specific analysis in refining the SNP-disease associations in PheWAS. In summary, this study provides a powerful approach towards the understanding of the functional associations in PheWAS and GWAS in terms of their functional mechanisms on affecting multiple complex diseases and traits.

## Conclusions

In this study, we proposed an integrative functional genomics framework that maps 215,107 significant SNP traits generated from the PheWAS Catalog and 28,870 genome-wide significant SNP traits collected from the GWAS Catalog into a global human genome regulatory map. By incorporating various functional annotation data from four major functional genomics databases—FANTOM5, ENCODE, NIH Roadmap, and GTEx—we showed that the disease-associated loci in both the PheWAS and GWAS Catalogs were significantly enriched with functional SNPs. We demonstrated that integration of functional annotations significantly improves the power of detecting novel associations in PheWAS and we further found a number of functional associations with strong regulatory evidence in the PheWAS Catalog. Furthermore, we performed a tissue-specific regulatory circuit analysis through integrating the identified regulatory variants and tissue-specific gene expression profiles in 7051 samples across 32 tissues from GTEx. We uncovered several promising tissue-specific regulatory TFs or genes for AD (e.g. ZIC1 and STX1B) and asthma (e.g. CSF3 and IL1RL1) in our case studies. In summary, this study offers powerful functional genomics tools and network methodology for exploring the functional consequences of variants generated from genome–phenome association studies in terms of their mechanisms on affecting multiple complex diseases and traits.

## Additional files

Additional file 1: Table S1. List of PheWAS SNPs located at ligand binding sites. (XLSX 9 kb)

Additional file 2: Table S2. List of PheWAS SNPs located at phosphorylation sites. (XLSX 9 kb)

Additional file 3: Table S3. List of GWAS SNPs located at ligand binding sites. (XLSX 9 kb)

Additional file 4: Table S4. List of GWAS SNPs located at phosphorylation sites. (XLSX 12 kb)

Additional file 5: Table S5. List of SNPs in different types of functional categories. (XLSX 546 kb)

Additional file 6: Figure S1. SNP annotation and enrichment analysis in different types of functional data. **Figure S2.** A colon-specific TF-target gene regulatory network for inflammatory bowel disease. (PDF 1073 kb)

## Abbreviations

eQTLs: Expression quantitative trait loci
GTEx: Genotype-Tissue Expression
GWAS: Genome-wide association study
ICD: International Classification of Disease
LD: Linkage disequilibrium
PheWAS: Phenome-wide association study
SNPs: Single nucleotide polymorphisms
TF: Transcription factor
