# Supplementary material for: An integrative functional genomics framework for effective identification of novel regulatory variants in genome–phenome studies
Source: Genome Med. 2018 Jan 29;10:7. doi: 10.1186/s13073-018-0513-x (PMC5789733; doi:10.1186/s13073-018-0513-x)
Supplement: Supplementary file 6 — SNP annotation and enrichment analysis in different types of functional data. Figure S2. A colon-specific TF-target gene regulatory network for inflammatory bowel disease. (PDF 1073 kb) [file 13073_2018_513_MOESM6_ESM.pdf]

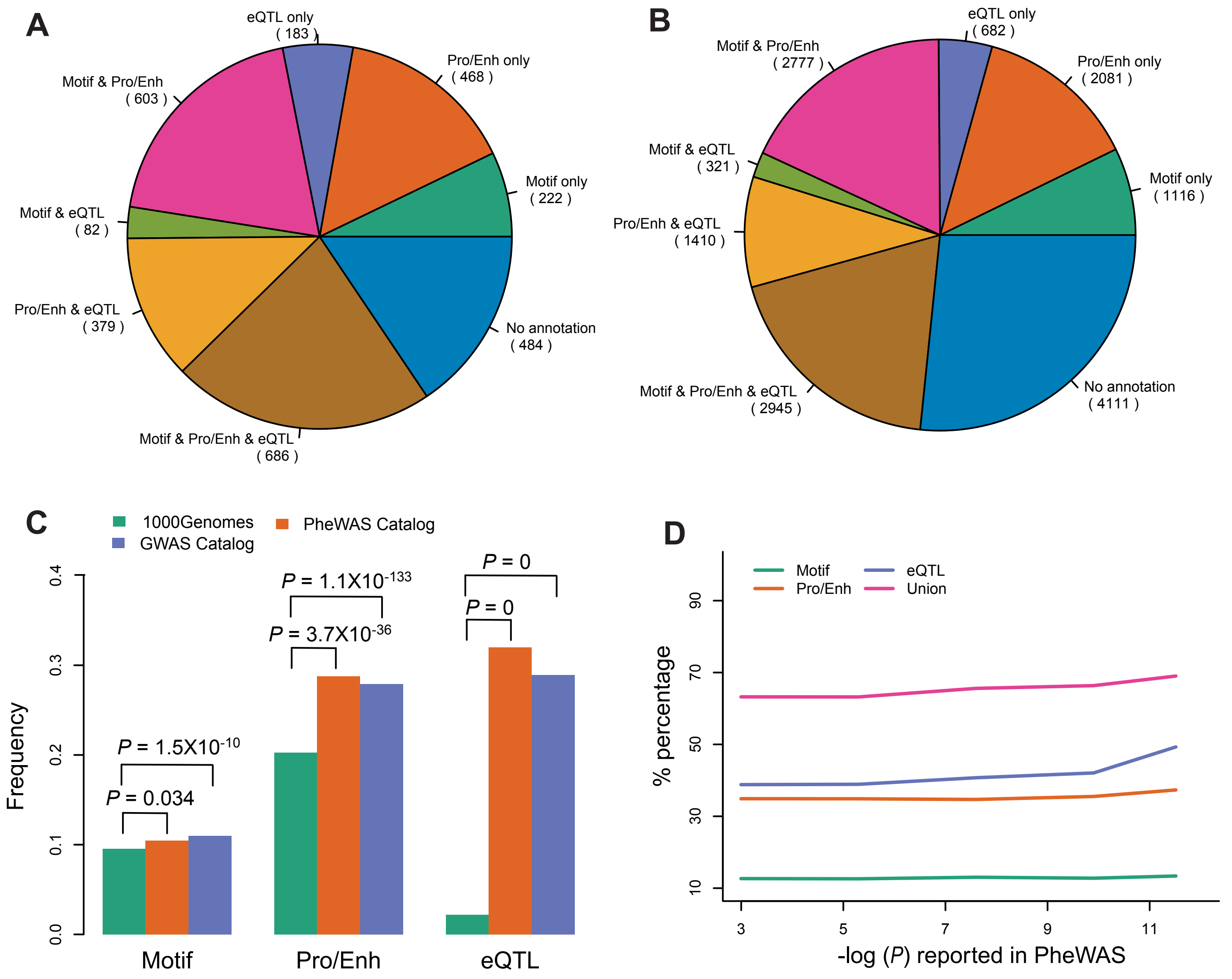

Figure S1. SNP annotation and enrichment analysis in different types of functional data. (A) Proportions of SNPs from PheWAS Catalog after applying different types of functional data with linkage disequilibrium (LD) extension. (B) Proportions of SNPs from GWAS Catalog in different types of functional data after LD extension. (C) Enrichment analysis of different types of functional data using the variants from the 1000 Genomes project as the background after LD extension. (D) Proportions of SNPs in different types of functional data by the P-value reported in PheWAS Catalog.

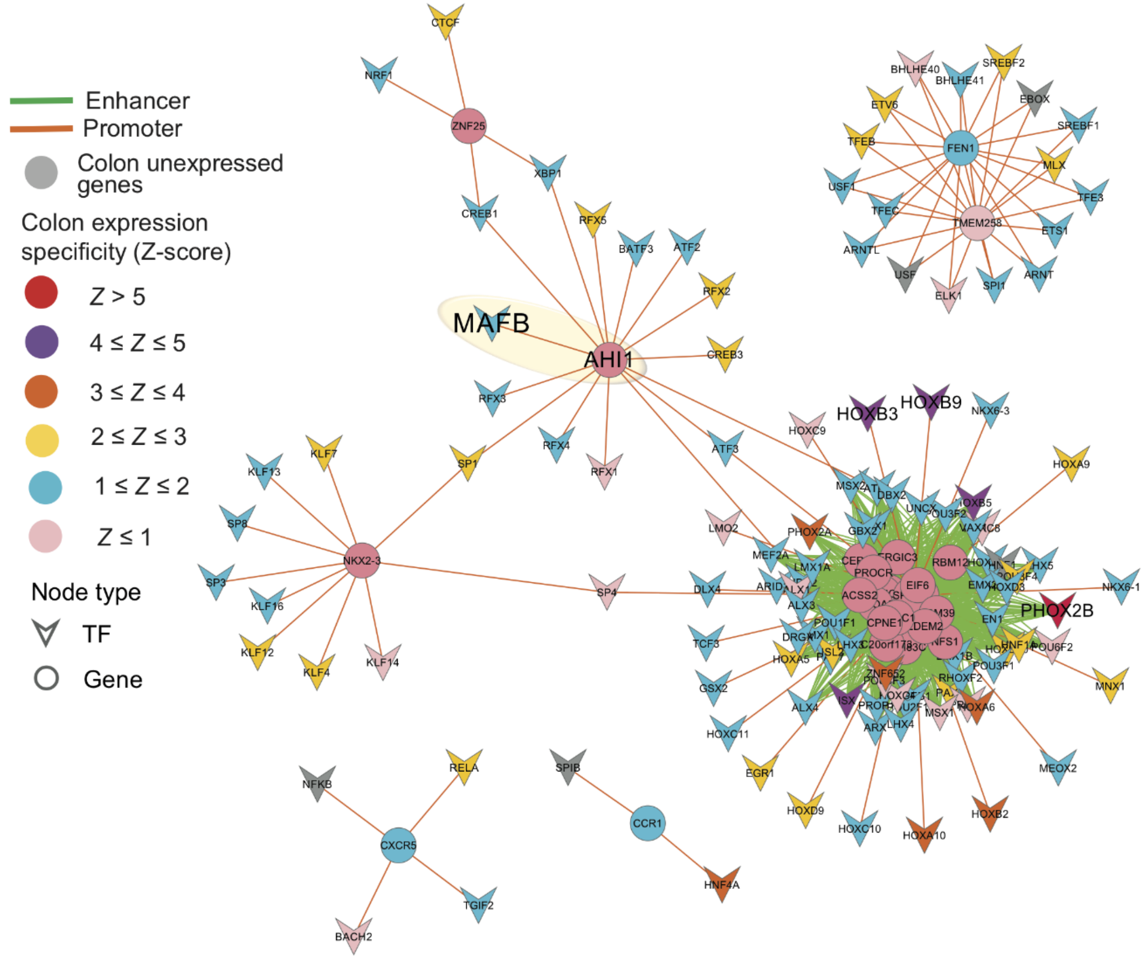

Figure S2. A colon-specific TF-target gene regulatory network for inflammatory bowel disease (IBD). TF denotes transcription factor. The TF-target gene network was generated by mapping the significant SNPs with IBD from PheWAS Catalog into the enhancer or promoter regions via three components: (1) mapping IBD-associated SNP to colon-specific promoter and enhancer regions, (2) linking TFs to promoters and enhancers, and (3) linking promoters and enhancers to target genes as described in Methods. Shape Vee denotes TF and circle denotes the target gene having the significant IBD-associated SNP. The node color is coded based on the colon-specific gene expression quantified by z-score using the RNA-seq data from GTEx (see Methods). A larger z-score indicates a higher expression level in colon compared to other tissues. We showed molecular validation of one of the network-predicted genes, *MAFB*, using recently published available functional data (Peters LA, et al. Nat Genet. 2017; 49:1437-49). *MAFB* encodes a transcription factor controlling macrophage self-renewal relevant to colitis (Soucie et al. Science 2016;351:aad5510). Primary human monocyte-derived macrophages treated with non-targeting, control small interfering RNA (siRNA) versus siRNAs targeting *MAFB*, under lipopolysaccharide (LPS) stimulation. We found that LPS stimulation yielded the largest differential expression signatures. Furthermore, siRNA-mediated knockdown of *MAFB* in macrophages significantly changed cytokine expression (MCP3 [CCL7], p-value = 0.0001) level. The detailed description of functional validation of *MAFB* in IBD was provided in a recent study (Peters LA, et al. Nat Genet. 2017; 49:1437-49).
